# Effects of the Menstrual Cycle and Hormonal Contraceptive Use on Metabolic Outcomes, Strength Performance, and Recovery: A Narrative Review

**DOI:** 10.3390/metabo14070347

**Published:** 2024-06-21

**Authors:** Hannah E. Cabre, Lacey M. Gould, Leanne M. Redman, Abbie E. Smith-Ryan

**Affiliations:** 1 Reproductive Endocrinology and Women’s Health Laboratory, Pennington Biomedical Research Center, Baton Rouge, LA 70808, USA; leanne.redman@pbrc.edu; 2 Independent Researcher, Advance, NC 27006, USA; 3 Human Movement Sciences Curriculum, Department of Exercise and Sport Science, University of North Carolina at Chapel Hill, Chapel Hill, NC 27599, USA; abbsmith@email.unc.edu

**Keywords:** eumenorrheic, monophasic oral contraceptive, intrauterine device, luteal phase, follicular phase, performance

## Abstract

The effects of female sex hormones on optimal performance have been increasingly recognized as an important consideration in exercise and sport science research. This narrative review explores the findings of studies evaluating the effects of menstrual cycle phase in eumenorrheic women and the use of hormonal contraception (oral contraceptives and hormonal intrauterine devices) on metabolism, muscular strength, and recovery in active females. Ovarian hormones are known to influence metabolism because estrogen is a master regulator of bioenergetics. Importantly, the menstrual cycle may impact protein synthesis, impacting skeletal muscle quality and strength. Studies investigating muscular strength in eumenorrheic women report equivocal findings between the follicular phase and luteal phase with no differences compared to oral contraceptive users. Studies examining recovery measures (using biomarkers, blood lactate, and blood flow) do not report clear or consistent effects of the impact of the menstrual cycle or hormonal contraception use on recovery. Overall, the current literature may be limited by the evaluation of only one menstrual cycle and the use of group means for statistical significance. Hence, to optimize training and performance in females, regardless of hormonal contraception use, there is a need for future research to quantify the intra-individual impact of the menstrual cycle phases and hormonal contraceptive use in active females.

## 1. Introduction

Although the potential impact of the menstrual cycle phases on athletic performance and recovery has recently garnered more research interest [1,2,3], the potential effect of hormonal contraception (HC; oral contraceptives and hormonal intrauterine devices) use has received limited attention. The impact of synthetic sex hormone administration on exercise physiology cannot be overlooked; over 60% of US adult women [4] and 49.5% of female athletes utilize some form of HC [5], with oral contraceptive (OC) pills and intrauterine devices (IUD) being the most common forms utilized [4,5]. However, many previous research studies omit HC users or have failed to accurately describe the reproductive status of the sample. Oral contraceptives introduce varying concentrations of circulating synthetic (external) estrogen and progesterone, which may influence physiological adaptations to exercise differently than endogenous sex hormones (hormones produced in the body). The impact of IUDs in the context of optimizing female performance may be similar to a eumenorrheic (EUM) menstrual cycle, as IUDs do not suppress endogenous hormone production. Yet, to our knowledge, there are only two previous studies providing an assessment of exercise performance in females utilizing IUDs [6,7].

Estrogen has been shown to aid in stimulating muscle repair and regenerative processes, including the activation and proliferation of satellite cells. Further, the presence of estrogen receptors on the mitochondria suggests a role of estrogen in the regulation of cellular bioenergetics [8,9,10]. As such, variations in estrogen concentrations across the EUM menstrual cycle phases, or via synthetic influence, may impact substrate utilization and skeletal muscle performance and repair in humans [11,12]. In animal models, 24 weeks of estrogen deficiency resulted in a 10% decrease in strength that corresponded with an 18% decrease in muscle cross-sectional area, demonstrating that estrogen may support greater force generation and skeletal muscle mass [8]. While pre-clinical studies have validated the potential of estrogen to improve muscle strength and maintenance [10,13], research in humans is less clear. Several human studies have demonstrated no significant differences in muscle strength between the low hormone follicular phase (FP), and the high hormone luteal phase (LP) [14,15], although the data are conflicting [16,17]. Additionally, the recovery and reconstruction of the muscle have been shown to be quicker in the mid-FP compared to the LP after eccentric exercise [18], possibly due to greater satellite cell activation in the mid-FP [19], yet there is no consensus on whether there is a relationship between hormone changes and time to recovery.

### 1.1. Aims and Methods of the Narrative Review

The recent publication of four extensive systematic reviews and meta-analyses examining the influence of female sex hormones on exercise performance [1,20,21] and the inclusion of females in exercise and sport science research [22] have highlighted the need to comprehensively examine the available studies evaluating the role of the menstrual cycle and hormonal contraception in strength and recovery for exercise performance. The heterogeneity in methodology and the definitions regarding EUM menstrual cycle phases and OC and IUD hormone phases have been highlighted as limitations within these meta-analyses. These limitations extend to comparing findings between EUM females and HC users, which have yet to be evaluated in a systematic review and meta-analysis. Narrative reviews consider the diversity of methodologies, participant characteristics, and outcome measures, allowing for a synthesis of the current literature to identify a future direction and gaps in the current literature.

In this review, we provide a brief overview of the EUM menstrual cycle and the most common methods of HC. We then detail how the physiology of the EUM menstrual cycle and HC impact metabolism, muscle strength, and recovery. We review how performance varies between EUM and HC users and across hormone cycle phases. Finally, we call for further research examining the inter-individuality of hormonal responses in association with performance, as a method to establish a consensus regarding the impact of the EUM menstrual cycle and HC use in female athletes.

A literature search of peer reviewed articles was performed using PubMed and Google Scholar to identify relevant articles to include in this review. Studies were reviewed if they examined recreationally active to elite athletes and reported menstrual cycle status, hormonal contraception use, and menstrual cycle phase study measurements. Muscular strength and power were delineated from anaerobic performance for the purpose of this review. As recovery is a dynamic process, we chose to include a wide variety of measures to globally assess the impact of EUM menstrual cycles and hormonal contraception use.

### 1.2. Overview of the Eumenorrheic Menstrual Cycle

**Figure 1:**
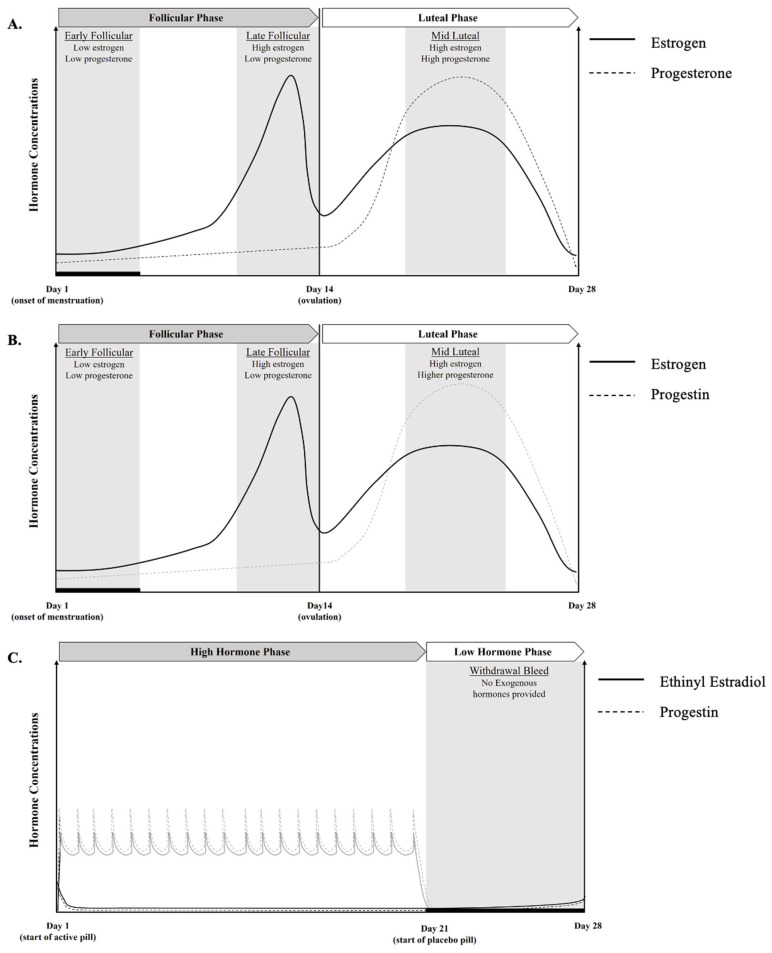
Visual representation of hormonal fluctuations across the menstrual cycle for an idealized 28 day cycle in (**A**) eumenorrheic cycles and (**B**) hormonal intrauterine device cycles, and hormonal fluctuations for (**C**) monophasic oral contraceptive users with a daily dose of synthetic hormones, which suppresses endogenous hormonal levels, as indicated by solid (estrogen) and dashed lines (progesterone).

Male and female physiology is similar until puberty, at which point sex hormones diverge. Once the transition through puberty is complete, females typically experience a circamensal rhythm, termed the menstrual cycle, that is characterized by predictable rhythmic fluctuations in four female sex hormones: estrogen, progesterone, follicle-stimulating hormone (FSH), and luteinizing hormone (LH) [23]. A regular EUM menstrual cycle can range from 23 to 38 days, and EUM females experience monthly menstruation until they reach menopause (cessation of menses) at ~51 years of age [23]. The menstrual cycle consists broadly of two phases: the FP and the LP, which are demarcated by ovulation (see [24] for a more detailed explanation of the menstrual cycle). Within the existing research, outcomes are most commonly compared between the FP and LP, with some studies evaluating ovulation as an additional timepoint [25]. The FP commenced on day 0, or the onset of menstrual bleeding. During the FP (days 0 to ~13), estrogen levels begin low, before rising to a peak 2–4 days prior to ovulation. Following ovulation or during the LP (days ~16 to 28), estrogen levels rise again, and progesterone peaks. If pregnancy does not occur, estrogen and progesterone levels fall until the next menses occurs (Figure 1) [24].

### 1.3. Overview of Common Hormonal Contraception Methods

Hormonal contraceptives provide systemic and localized synthetic female sex hormones. By acting on the hypothalamus and anterior pituitary glands, synthetic sex hormones suppress gonadotropin-releasing hormone, FSH, and LH, thus reducing endogenous estrogen and progesterone levels [26]. In both nonathletes and athletes, HC are commonly prescribed for cycle regulation, contraception, treatment of amenorrhea, and for the maintenance of bone density, amongst other reasons [27,28]. There are various delivery methods for HCs, including OC, implant, injection, transdermal patch, vaginal ring, and IUD, with OC and IUD being the most commonly prescribed HC in the United States [29]. Furthermore, HC can be classified by type: combined, with inclusion of both estrogenic (e.g., ethinyl estradiol) and progestin compounds, or progestin-only. The type and concentration of ethinyl estradiol and progestin varies between different HC types and may influence physiological responses to exercise.

In EUM females in the USA, the OC pill is most commonly prescribed, in the form of a combined ethinyl estradiol and progestin tablet that is consumed daily for 21 days, followed by a 7-day inert pill (called a sugar pill) to allow for monthly bleeding [30]. However, the bleeding that occurs with OCs is not considered a true period [30]. The combined pill can provide a monophasic, biphasic, or triphasic dosage pattern, with each providing a varying release of hormones across a 21-day period [27]. Monophasic OC are the most common OC pill prescribed and researched [25,30]. Of the various forms, monophasic OC has been thought to be beneficial for the easy manipulation of the menstrual cycle and less breakthrough bleeding for the treatment of dysmenorrhea, and an effective contraception [20,27]; thus, it will be the focus of this review. Monophasic OC contains doses of ethinyloestradiol and progestins that are delivered at a constant dose over 21 days, followed by 7-day placebo pill to allow for monthly bleeding (Figure 1). Other OC pills that are prescribed include a triphasic pill in which the dose of ethinyl estradiol and progestin change three times in a 21-day period [27], and a biphasic pill in which the progestin level is increased halfway through the pill cycle, while the level of estrogen stays consistent [27]. The main drawback of triphasic pills for training and performance is difficulty with cycle timing, due to the variations in synthetic hormone concentrations throughout the doses [27], while the biphasic form is the least popular and offers no particular advantage over the other two forms.

IUDs are another form of hormonal contraceptives that are increasing in usage. IUDs are long-acting contraceptives placed directly into the uterus that provide consistent menstrual cycle manipulation for three to five years. There are two forms: a copper IUD that does not contain synthetic hormones, and an IUD containing progestin. Copper IUDs are strictly for contraception, and since they do not provide any synthetic hormones, have less menstruation symptom management benefits. For progestin IUDs, there are two types: one containing 52 mg of Levonorgestrel (progestin), which can last for five years, and the other containing 13.5 mg Levonorgestrel, which lasts for three years [31]. Both administer the progestin locally into the uterus (i.e., endometrium), which does not completely suppress endogenous hormone production by the hypothalamic-pituitary–gonadal axis [32]. Hormonal IUDs act by thickening the cervical mucus, causing endometrial decidualization and glandular atrophy, which inhibits sperm adhesion to the ovum and the partial inhibition of ovulation [31]. Hormonal IUDs are often prescribed to help manage menstruation symptoms and can include a possible loss of menstruation (i.e., dysmenorrhea). The use of IUDs may be similar to EUM menstrual cycles, as the lack of a systemic hormonal interference results in a similar physiological response pattern to that of a EUM cycle (Figure 1) [6].

**Figure 2:**
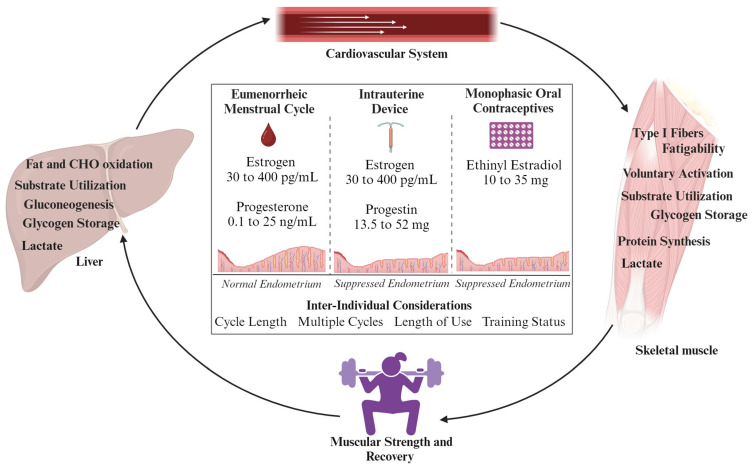
Overview of physiological systems related to muscular strength and performance that may be directly or indirectly impacted by estrogen levels, and variations in hormonal profiles between eumenorrheic females and those using hormonal contraception. Both intrauterine devices and monophasic oral contraceptives suppress the endometrium through progestin, which can also indirectly impact metabolism and skeletal muscle [34]. Future considerations should examine inter-individual factors that impact related physiological systems, strength, and recovery over time.

While the primary role of female sex hormones are reproductive actions, they are known to impact other physiological systems, such as the respiratory, thermoregulatory, cardiovascular, and bioenergetic systems [10,33]. Taken together, the diverse hormonal profiles between EUM and HC methods may impact these systems differently, which holds important considerations for active females. This is particularly true regarding metabolism and skeletal muscle mass performance. Henceforth, this review seeks to collate data regarding the endogenous and synthetic effects of key physiological systems related to muscular strength and recovery (Figure 2).

## 2. Defining Metabolic Outcomes in Females: Key Hormonal Considerations

Metabolism is an important determinant for nutritional status and has important implications regarding exercise performance. The fluctuations of female hormones across a EUM menstrual cycle facilitate variations in energy expenditure and macronutrient metabolism [23]. Substantial evidence suggests that estrogen is a master regulator of bioenergetics in females [10]. The impact of an EUM menstrual cycle phase on metabolism and performance has been well evaluated [12,23,35,36,37].

### 2.1. Substrate Utilization and Female Sex Hormones

The mechanisms by which estrogens influence substrate metabolism and utilization are due to the direct and indirect actions of the hormone. Estrogens directly impact metabolism through lipolytic enzymes, which regulate the mobilization of stored fatty acids in the adipose and muscular tissue, subsequently influencing carbohydrate utilization [38]. A recent review demonstrated fat oxidation capacity is maximized in the late FP and mid-LP due to estrogen signaling mechanisms [34]. Additionally, estrogen promotes glucose uptake during exercise by stimulating GLUT4 expression and kinases. Indirectly, estrogens mediate other hormones, such as cortisol, that promote lipolysis and glycogenesis at rest and limit glycogenolysis during exercise [38,39,40]. A general consensus of previous research suggests there is a decrease in fat oxidation, protein oxidation, and glycogen storage, with an increase in carbohydrate oxidation in the FP [12,23]. During the LP, there is an increase in fat oxidation, protein oxidation, and glycogen storage, with a decrease in carbohydrate oxidation [23]. These variations in substrate utilization across the menstrual cycle hold important implications for strength performance, particularly as the skeletal muscle stores of ATP (energy) are small [41]. Thus, the ability to stimulate muscle glycogen breakdown for substrate-level phosphorylation and oxidative phosphorylation (carbohydrate and fat metabolism) is imperative for performance and glycogen balance during recovery. Higher concentrations of estrogen have resulted in higher fat oxidation and lower carbohydrate oxidation during aerobic exercise [23,39], yet the exploration of substrate utilization during anaerobic exercise, and in HC users, is limited.

### 2.2. Resting Energy Expenditure and Female Sex Hormones

In addition to the impact of female sex hormones on substrate utilization, estrogen and progesterone also impact resting energy expenditure. Recent works demonstrate estrogen can increase the pituitary gland to secrete key hormones that regulate the metabolic rate, such as thyroid hormones and growth hormone [42,43]. At rest, EUM females exhibit heightened fat oxidation, as indicated by a decreased respiratory exchange ratio, and a 2.5–11.5% higher resting energy expenditure during the LP of the menstrual cycle when ovarian hormones peak [44,45]. Supporting this, a recent meta-analysis examining 26 studies found there was a small but significant effect favoring increased RMR in the LP (effect size = 0.33; 95% CI = 0.17, 0.49, *p* < 0.001) [46]. One study, evaluating resting energy expenditure in EUM females during the mid-LP (elevated estrogen and progesterone), the early FP (low estrogen), and after 6 days of estrogen and progesterone suppression, demonstrated that resting energy expenditure tended to be greater in the mid-LP, when estrogen and progesterone were elevated, than during the suppression of estrogen and progesterone [47]. Resting energy expenditure and energy intake are the components of the energy balance equation, which can dictate if an individual is in a positive or negative energy balance. Therefore, accounting for energy expenditure in female athletes is an important component of nutritional strategies, especially when aiming to prevent low energy availability [48]. Resting energy expenditure was the highest in the mid-LP, lower (−29 kcal/d) in the early FP, and further reduced (−42 kcal/d) after estrogen and progesterone suppression [47]. These results suggest that elevated estrogen and progesterone support an increased energy expenditure in females, and that resting energy expenditure may vary across the EUM menstrual cycle. While these mean differences may not surpass the measurement error, they demonstrate that hormonal fluctuations may impact energy expenditure, which is an important consideration for recovery and proper nutrient intake. Additionally, the data herein suggest that there is inter-individuality between measures, highlighting the importance of individual considerations regarding hormonal status. Currently, only six studies have investigated the effect of varied OC types on resting energy expenditure, with inconsistent results. One study, that did not control for the hormone phases, showed an increase in resting energy expenditure (+46 kcal/d) with contraception use [49].When EUM women were compared to OC users (FP compared to withdrawal pill and LP compared to active pill), five studies showed no differences in resting energy expenditure or basal metabolic rate between groups [50,51,52,53,54]. Due to the heterogeneity of the few available studies (i.e., study population, study design, OC formulation and dose), the influence of HC use on resting energy expenditure is unclear.

### 2.3. Protein Synthesis and Female Sex Hormones

With regards to strength, skeletal muscle growth and repair are paramount. Protein turnover, the rate of muscle protein synthesis and breakdown, determines the anabolic or catabolic state of skeletal muscle and is influenced by protein oxidation [55]. In turn, recent evidence suggests estrogen reduces protein oxidation, while progesterone, which peaks in the LP of the menstrual cycle, may elicit catabolic effects [24,56,57]. Of the limited data evaluating protein turnover across the menstrual cycle in healthy EUM females, studies with small sample sizes have consistently found phenylalanine, lysine, and leucine oxidation are greater in the LP compared to FP [58,59,60]. This suggests elevated progesterone may increase the oxidative disposal of amino acids [23], possibly facilitating a systematic catabolic environment in the LP. Reports on amino acid flux and net balance are less consistent, highlighting the need for a further evaluation of protein turnover in various hormonal profiles, particularly as alterations in protein balance may dictate changes in lean mass [56]. Research examining the anabolic functions related to estrogen suggests increased concentrations of estrogen, coupled with increased muscle protein synthesis via exercise stimulation, can improve skeletal muscle performance [26]. In EUM females undergoing a bout of one-legged exercise once in the FP and once in the LP, there were increases in myofibrillar and muscle collagen protein synthesis (measured by the incorporation of [(13)C]leucine) at 24 h postexercise in both phases, with no differences between phases [58]. When compared to EUM, OC users had a lower myofibrillar protein fractional synthesis rate in the fed state, although the difference seemed to depend on progestin in the OC type. Interestingly, exercise did not change the myofibrillar protein fractional synthesis rate in either group [59]. Understanding the protein turnover response to the fluctuating hormone concentrations across the menstrual cycle is essential for protein and recovery recommendations in female athletes. To date, we are not aware of any data evaluating the effects of various HC phases or IUD use on protein turnover.

## 3. Defining Muscular Strength and Power in Females

Adequate levels of muscular strength and power are needed for physical functioning, reduced risk of injuries, and optimal performance in female athletes [61,62]. Skeletal muscle characteristics influence exercise performance through energy provision, resistance to fatigue, and the ability to adapt to exercise. Sex differences occur in skeletal muscle mass, particularly as males possess a greater quantity of skeletal muscle than females, contributing to greater strength [63,64]. Additionally, skeletal muscle is more sensitive to the male anabolic hormone, testosterone, than the anabolic hormones in females, estrogen, leading to sex differences in training and performance [65]. However, females typically have a greater proportional area of type I muscle fibers, thereby incurring greater capillarization, mitochondrial respiratory capacity, and fatigue resistance compared to males [63]. The greater proportion of type I fibers and estrogen concentrations affects muscle metabolism during exercise, with females oxidizing more fat but less carbohydrates and amino acids compared with males [66]. The proportional fiber-type difference between sexes also influences the contractile properties of skeletal muscle in males and females.

The contractile apparatus of the skeletal muscle motor pathway, composed of actin, myosin, and other proteins, is ultimately responsible for force generation and human movement [67]. Muscle contractions are initiated by the central nervous system (CNS), and muscle activation is a limiting factor in exercise performance, with changes in neurotransmitter concentrations and motoneurons influencing the ability to drive the muscle [4,68]. Previous data suggest that, while the voluntary activation of muscular contraction does not differ between sexes [63,69], the fluctuations of estrogen and progesterone across the EUM menstrual cycle can affect CNS function due to their ability to cross the blood–brain barrier [70]. Specifically, a recent investigation demonstrated that voluntary activation was greater when estrogen levels peaked (days 9–11 of the menstrual cycle and/or approximately 3–5 days prior to ovulation) and lower when progesterone levels peaked (+7d post ovulation) in EUM females [71]. This suggests that the EUM menstrual cycle may impact neuromuscular function and strength. Yet, previous studies evaluating the EUM menstrual cycle effects on voluntary activation and motor function are equivocal, with studies demonstrating 8–23% greater maximal voluntary contraction force during midcycle (late FP) [16,72,73].

Muscular power is a major factor for evaluating injury risk and sports performance, especially in sports that are dependent on high-speed movements [74]. The mechanical power of jump movements depends on neuromuscular coordination and the restitution of elastic energy stored in muscles and tendons. Previous research has demonstrated significant variations in knee joint laxity, neuromuscular coordination, and postural control during the menstrual cycle, with greater laxity occurring around ovulation (late FP) [75,76,77]. Estrogen receptors are widely distributed in skeletal muscle, tendons, and mitochondria, so variations in estrogen concentrations, naturally or via synthetic hormonal influence, may impact skeletal muscle performance and repair [11]. Given the fluctuations of sex hormones across the menstrual cycle, skeletal muscle performance after exercise may differ in response to variations in estrogen and progesterone levels. Thirteen studies evaluating muscle strength during different phases of the menstrual cycle, and some between EUM to OC users, demonstrated that there are equivocal differences in muscle strength during different phases of the menstrual cycle [14,15,16,26,76,78,79,80,81] (Table 1). Interestingly, studies that compared EUM and OC users demonstrated no significant differences or changes between cycle phases [26,76,77,78,79]. This section will evaluate studies that confirmed the menstrual cycle phase to examine if there are differences in strength outcomes. Generally, it appears that maximal strength, isokinetic dynamometry, and time to task failure were improved in the LP compared to FP or ovulation [71,76,81]. However, in the limited body of literature, studies often do not clearly present their methodology in menstrual cycle determination [25] or present the mean values neglecting the intra-individual variation that may occur [1,20]. While this overgeneralization in the existing reported data may not overtly impact recreationally active females, it may have consequences for elite female athletes, whose training and performance can be challenged by muscular strength differences resulting from menstrual cycle fluctuations or hormonal contraceptive use. To date, there is minimal research in recreationally active and elite athletes investigating the intra-individual EUM menstrual cycle-induced changes in strength, with the impact of OC or IUD use on strength being even less understood. 

### 3.1. Maximal Strength and the Hormone Cycle

One repetition max (1RM) is defined as the maximal weight that can be lifted at once while maintaining the correct lifting technique, and it allows for assessing strength in multi-joint exercises [74]. While 1RM tests are considered the “gold standard” for assessing dynamic strength, most commonly used muscle strength tests in previous studies, which do not focus on female athletes, have included a handgrip test and isokinetic testing for knee flexors and extensors. There is limited research evaluating the effect of varied hormonal profiles on 1RM strength. Romero-Moraleda et al. [15] sought to investigate the fluctuations of female sex hormones across the EUM menstrual cycle on muscle performance using the Smith machine half-squat exercise in 13 female triathletes. The results demonstrated no significant differences in 1RM during the early FP (mean ± standard deviation: 97.0 ± 23.2 kg), late FP (98.5 ± 18.1 kg), and mid-luteal phase (98.1 ± 22.2 kg), suggesting that there are no systematic variations in muscle performance during the different EUM menstrual cycle phases [15]. The study by Myllyaho and colleagues [78] examined the effects of HC use on 1RM leg press, demonstrating similar strength between HC users (mean ± standard deviation: 114 ± 15 kg) compared to EUM females (118 ± 18 kg) [26]. A similar trend was observed for 1RM bench press values between HC users and EUM (43 ± 8 kg and 41 ± 10 kg, respectively). However, these two studies did not study participants while they were in the same hormonal phase, or standardize the HC used. Hence, our understanding of hormonal influences on 1RM strength is grossly limited. The scarcity of research regarding maximal strength output with different hormonal profiles highlights the necessity of understanding the differences between HC types and hormone phases on maximal strength to accurately prescribe training recommendations to active females. 

### 3.2. Isometric Dynamometer and the Hormone Cycle

Muscular strength is most commonly assessed with force measurements from static or dynamic muscle contractions, as they can objectively measure static muscle strength and force production, two key aspects of power development [83]. Isometric dynamometry also measures motor function, which is important to evaluate in female athletes, as the voluntary activation of muscular contraction may differ in response to female sex hormones [71]. Several authors have concluded that there are no significant differences in maximal force production during different phases of the menstrual cycle [14,26,79,82], although there are conflicting data [16,81]. Early research evaluating the isometric strength of the quadriceps across the EUM menstrual cycle found that there was a peak in strength around the time of ovulation [81]. This observation was supported by another study, reporting an 11% increase in quadriceps maximum voluntary isometric force in the EUM ovulation period compared to early FP or LP [16]. However, females using OC in the study demonstrated no changes in quadriceps maximum voluntary isometric force, suggesting that a peak in estrogen may have contributed to the observed changes in isometric strength. Since these early investigations, research regarding isometric strength across the menstrual cycle has been equivocal, possibly due to the various methods used to estimate the phase of the EUM menstrual cycle and the different definitions of reproductive status. When comparing monophasic OC users to EUM females undergoing 60% of maximal voluntary contraction with the knee extensors until task failure, voluntary activation peaked for both groups in the FP with no effect for HC use [71]. In another study comparing HC users to EUM females, isometric leg extension force was similar between groups (HC: 2840 ± 405 N and EUM: 2680 ± 631 N, respectively), suggesting synthetic hormones may not influence isometric strength [26]. However, again this study did not standardize testing to a hormone phase or type of HC. Furthermore, when OC users were compared to EUM females, there were no significant differences in the maximal voluntary force of the quadriceps between groups, despite a significant increase in the concentration of progesterone and estrogen in the LP for EUM, and the active pill for OC compared to FP and withdrawal [79]. The research regarding isometric strength in different hormone phases and with different HC types remains limited.

### 3.3. Muscular Power and the Hormone Cycle

Muscle power is a major factor for evaluating injury risk and sports performance, especially in sports including high-speed moments [74]. Common methods for assessing anaerobic power include the Margaria–Kalamen staircase test, Wingate cycle test, and maximal sprints to exhaustion. While these methods can also infer muscular power, drop jumps and maximal vertical jumps are more sensitive methods to evaluate muscular power, as they assess reactive strength, the stretch-shortening cycle utilization, and the neuromuscular control. Of the available research assessing these methods of muscular power in EUM females and HC users, there appears to be no difference between groups or hormone phases on muscular power [76,77,80]. When evaluating the effect of the hormone cycle phase on maximal jump height in OC users compared to EUM, there were no differences between groups or between hormone cycles for maximal jump height [77]. Another study, observing monophasic OC use on a 45 cm drop jump, reported lower muscular power occurring during the withdrawal phase compared to the active phase in trained athletes [80]. Considering that variations in estrogen levels may influence knee joint laxity and neuromuscular coordination [75,76,77], evaluating the inter-individual responses of hormone cycle phases and various hormonal profiles across multiple menstrual cycles will aid in elucidating the implications female sex hormones have for muscular power, if any.

## 4. Overview of Recovery in Females

For exercise performance, it is essential to be able to perform, but also to recover rapidly from high-intensity bouts of exercise. Recovery is a critical period relative to the adaptation process during training regimes, and is associated with an adequate balance between training load and rest, which is essential for exercise adaptations [84]. The relationship between fitness (i.e., a positive outcome of training) and fatigue (i.e., a negative result of training) is important for exercise performance and adequate recovery. Higher levels of fatigue can predispose individuals to injury or overreaching. Importantly, there are sex-based differences regarding fatigue, with evidence demonstrating that female skeletal muscle is more resistant to fatigue than male skeletal muscle when undergoing equivalent dosages of high-intensity exercise [63]. Historically, trained females have exhibited faster oxygen uptake kinetics during moderate-intensity exercise and smaller decreases in ATP concentrations after all-out exercise compared to males [85,86]. A recent investigation comparing recreationally males and females (no control for menstrual cycle phase or OC use) demonstrated similar relative values of VO_2_ gain and %VO_2peak_ between sexes. Baseline VO_2_ was slightly elevated at the onset of heavy-intensity exercise compared to moderate- intensity exercise in females, while males demonstrated no baseline differences [87]. A consistent finding in previous research comparing males and females exercising at the same metabolic intensity is that females experience a lesser degree of contractile impairment of the knee extensors [88,89,90]. As such, female skeletal muscle may be more efficient at resynthesizing ATP from oxidative phosphorylation during high-intensity exercise, which has particular implications for decreased fatiguability and increased exercise recovery [69]. Therefore, it is possible that females require less rest between intense interval exertions for adequate metabolic recovery.

Effective recovery strategies have demonstrated that an increased blood flow to the recovering muscles may improve oxygen and nutrient delivery, and therefore the resynthesis of phosphocreatine and glycogen [91,92]. The perfusive and hemodynamic properties of female skeletal muscle have indicated improved oxygen and metabolite removal during and after exercise, aiding in reduced fatigue during exercise and increased recovery post-exercise [63]. Elevated estrogen levels in females have been shown to promote vasodilation, by stimulating nitric oxide synthesis and decreasing the production of vasoconstrictor agents [33]. Increased vasodilatory responses, paired with a higher density of capillaries per unit of skeletal muscle, provide greater blood flow to exercising skeletal muscle. This possibly decreases the metabolic perturbations that induce fatigue [69,85,93,94]. Increased blood flow may aid in transporting lactate and other metabolic by-products away from the active muscle to removal sites, possibly increasing the blood lactate threshold during exercise and expediating lactate clearance post-exercise [95]. However, there is limited research characterizing blood lactate levels or clearance in both EUM females and those using HC.

Furthermore, estrogen may act to diminish skeletal muscle damage and inflammatory responses after exercise by exerting a protective effect on muscle membranes via direct receptor-mediated mechanisms, and is a bioactive in stabilizing plasma membranes and limiting free radicals [9,96]. This reduction in muscle membrane disruption may also be important for muscle repair and regeneration. Estrogen and progesterone have also demonstrated antioxidant-like characteristics through promoting the expression of antioxidant enzymes, which inhibits inflammatory responses post-exercise and attenuates exercise-induced muscle damage in the late FP and mid-LP, thereby accelerating recovery [9,96]. A recent review of the menstrual cycle effects on exercise-induced fatigability found fifteen studies with a statistical difference between the FP and LP in EUM menstrual cycles. Eight studies demonstrated greater fatigue resistance in the FP, and seven studies demonstrated greater fatigue resistance in the LP [2]. Additionally, there is a great diversity in the outcome measurements reported for measuring fatigue (e.g., biomarkers, blood lactate, blood flow). Twelve studies have assessed fatigue outcomes in EUM, OC users, and IUD users, with the results demonstrating equivocal differences between the cycle phases (e.g., FP, ovulation, LP, active pill, withdrawal bleed) and contraceptive types (Table 2). The inconsistencies across studies may be a consequence of the differences in methodologies and the techniques used to define the menstrual cycle phase (e.g., serum hormone concentration vs. reported day of menses). Therefore, an evaluation of the impact of changes in estrogen concentration across the menstrual cycle, or with synthetic hormone use, is important for characterizing recovery and fatigue in females.

### 4.1. Recovery and the Menstrual Cycle

Due to estrogen’s role in reduced muscle damage and inflammation after exercise, it is hypothesized that recovery might be enhanced in the late FP, when estrogen peaks. However, the current body of literature examining the recovery differences between EUM menstrual cycle phases provides equivocal evidence. Recovery is often measured by evaluating the concentration of circulating inflammatory markers, such as creatine kinase (CK) and interleukin-6 (IL-6), which are products of muscle cell myoblasts and satellite cells’ response to muscle injury. Early works reported that resting IL-6 levels are lowest in the LP when progesterone levels are elevated, and highest in the FP during normal menstruation, when estrogen and progesterone are low [108]. Recent works by Hackney, Kallamen, and Ağgön (2019) confirmed this observation in EUM active females, reporting elevated IL-6 values in the mid-FP at rest, immediately after running for 90 min at 70% VO_2_ max on a treadmill, and 24-h post-exercise (mean difference ± standard deviation: 1.4 ± 1.9 pg/mL, 24.9 ± 13.2 pg/mL, and 10.3 ± 7.1 pg/mL, respectively) compared to the mid-LP (1.2 ± 0.5 pg/mL, 13.5 ± 6.2 pg/mL, and 5.0 ± 3.0 pg/mL, respectively) [97]. In contrast, another study in well-trained EUM females following an eccentric exercise demonstrated a significant interaction for IL-6, indicating a possible inflammatory response in the mid-LP compared to mid-FP [98]. However, the participants were well-trained, and the exercise stimulus may not have induced sufficient muscle damage to fully observe recovery measures. Other works involving EUM females have demonstrated no differences in the blood markers of recovery between menstrual cycle phases [99,100]. Additionally, there have been limited studies utilizing other recovery outcomes, such as blood flow or blood lactate between menstrual cycle phases. Estrogen can mediate endothelial function, particularly the vasodilatory responses of the arteries, providing greater blood flow to exercising skeletal muscle [33,93]. Effective recovery strategies have demonstrated that an augmented blood flow may improve oxygen and nutrient delivery [91], possibly decreasing the metabolic perturbations that interfere with adaptation [93]. A recent meta-analysis evaluating the impact of the menstrual cycle phases on peripheral vascular function in premenopausal women found approximately 30 studies provided evidence that endothelial function increased in the LP [106]. Additionally, increased blood flow during exercise may aid in transporting lactate and other metabolic by-products away from the active muscle [91], increasing the blood lactate threshold and accelerating lactate clearance post-exercise; both of which have been correlated with improved performance [109]. One study examining blood lactate concentrations in EUM using repeated sprints on a treadmill found that a significant increase in blood lactate was observed over time, yet there were no significant differences between menstrual cycle phases (prior: Δ 0.01 mmol/L; during: Δ −0.8 mmol/L; immediately post: Δ −0.03 mmol/L; 10-min post: Δ −0.4 mmol/L) [101]. Other studies examining blood lactate values in EUM women have also demonstrated equivocal lactate accumulation after intense exercise, suggesting blood lactate may not vary across the menstrual cycle phase [3,102,103,104].

### 4.2. Recovery and Hormonal Contraception

Considering OC use downregulates the cyclic hormone activity of the hypothalamic-pituitary–gonadal axis, recovery outcomes in OC users may differ from EUM females. Some research has shown that the synthetic hormones of OC may modulate the recovery metrics, such as respiratory rate, sleep, and heart rate variability, in a different pattern than the EUM menstrual cycle, with reduced indices of adaptation to stress across pill types (progestin-only and combined) and phases [110]. One study on healthy, active females (EUM and OC users) demonstrated decreased recovery, as measured by heart rate variability (a surrogate marker for ANS activity) across the menstrual cycle, from the mid-FP to the mid-LP in EUM females [110]. Females utilizing combined OC in the same study demonstrated larger reductions in recovery with every unit of increased cardiovascular strain (e.g., decrease in heart rate reserve) compared to EUM or progestin-only pill users, regardless of cycle phase [110]. The authors suggested recovery in OC users may have been attenuated due to the greater inflammatory responses associated with OC use [111,112], particularly due to increases in free circulating cortisol in the blood often observed with combined OC [113]. In female collegiate soccer players utilizing OC, stress, inflammatory, and cortisol levels were elevated throughout a competitive season compared to EUM females, suggesting OC use may be pro-inflammatory, thereby leading to decreased recovery [114]. When observing workload and power decrements during a repeated sprint ability test, another marker of the capacity to recover, in the withdrawal week and active week of monophasic OC, team sport athletes demonstrated equivocal workload and power decrements during the withdrawal phase compared to the active phase [80]. However, the study did not utilize a comparator EUM group. A recent review evaluating the impact of hormonal contraception use, including OC and IUDs, on peripheral vascular function and structure, demonstrated that hormonal contraceptives appear to impact both macrovascular and microvascular endothelial function, with phasic differences in some contraceptive types depending on the progestin and route of administration. Interestingly, OC use appeared to impair endothelial function to a greater extent than IUD, when measured during the active pill and FP [107]. Regarding other markers of recovery, in a recent review evaluating the impact of OCs and IUDs on vascular function,, IUDs had no effect, while OC use resulted in a lower blood flow [107]. Another study examining blood flow and blood lactate concentrations in OC and EUM women found intense exercise appeared to blunt the skin blood flow mechanisms in the OC, but blood lactate concentrations were not different between groups [105]. A recent study found blood flow and blood lactate values between EUM, OC, and IUD were similar across hormone cycle phases [7]. While not significant, the OC group demonstrated reduced blood lactate clearance and greater fatigue compared to EUM and IUD groups. With conflicting findings, it is important to understand the implications HCs have on training adaptations and recovery.

## 5. Limitations and Future Considerations

Strength and recovery are imperative measures for optimal training and performance in active females [26,110]; evaluating the impact of female sex hormones on these outcomes poses a great opportunity for understanding of the role of the menstrual cycle and hormonal contraception in athletic populations. Recently, interest in female-specific sport and exercise science data has increased; however, there are key limitations to previous research that limit the translatability of findings into practice. One limitation that exists is evaluating training and performance outcome measures across only one menstrual cycle, and are evaluated in non-athletes or recreationally active females. A recent article combining data from several cohort studies examining patterns in menstrual cyclicity demonstrated a great diversity in cycle length. The variability in FP and LP length was substantial. Within-female FP length variability was greater than 7 days in 42% of females and within-female LP length variability was more than 3 days in 59% of females [115]. This introduces a limitation regarding the repeatability of outcomes. Therefore, studies should include repeated measures across multiple menstrual cycles to strengthen the evaluation of performance measurements in females. Furthermore, the single menstrual cycle approach may reduce the ability to accurately compare EUM cycles to OC or IUD cycles, due to the diversity in hormonal fluctuations between types and phases. This evaluation is further limited by a few studies including hormonal IUDs, despite their prevalence as a hormonal contraception method [6]. Future studies should consider including hormonal contraception comparator groups to provide insightful data reflective of the current hormonal landscapes of the general public [116].

Precision medicine is an emerging innovative approach to managing health and performance that considers individual differences in genes, environments, and training [117]. Female athletes represent a population that would greatly benefit from precision medicine, particularly related to training and nutrition. However, the current literature is limited by the use of standard statistical approaches, evaluating the mean as the primary determinant of statistical significance and summing each individual datapoint together. Relationships that exist among individuals may not be the same as those relationships that exist within individuals [118]. Using group means as standard practice does not allow for the evaluation of individual responses across the menstrual cycle or with hormonal contraception use, thereby limiting the translatability of the known diversity in hormonal profiles among each female. As such, even if there are no differences in group means, individuals will have marked differences between menstrual cycle phases that are lost in the analysis [115,119]. An analysis of the intra-individual responses in outcomes (e.g., strength and recovery) within study timepoints will provide a deeper understanding of how female sex hormones, both endogenous and synthetic, impact optimal training and performance. More data on intra-individual responses will provide a greater foundation for precision medicine, aiding in optimal strength performance, recovery, nutrition, and therapeutic benefits. This distinction is particularly important for elite female athletes who are expected to perform across all phases of the menstrual cycle.

## 6. Conclusions

This narrative review investigated the current state of the literature regarding the effects of the EUM menstrual cycle and HC use on metabolism, muscular strength, and recovery. This review provides an important exploration of how various female sex hormone profiles can influence metabolism and exercise performance, with the majority of the literature suggesting that when group means are evaluated, there are few differences between EUM and HC users and across hormone phases. There is a wide variety of methodology used to assess endogenous and synthetic female sex hormones, with few studies examining the impact of IUD use on strength and recovery. Future research should focus on the inclusion of HC use, which may expand female inclusion criteria in scientific research. Additionally, repeating measures across multiple menstrual cycles will increase longitudinal translatability, as training is a chronic stimulus. Furthermore, reporting intra-individual data will increase the longitudinal translatability and transparency within sport and exercise science research.

## Figures and Tables

**Table 1:** Summary of strength, isometric/isokinetic contractibility, muscular power, and neuromuscular observations in EUM females compared to HC users, within EUM females across the menstrual cycle, and within HC users during active and inactive pill phases. All studies included recreationally active to active females.

EUM Females Compared to Females Using HC					
Source	Outcome	EUM Females	Hormonal Contraceptive Users		n
Myllyaho et al. [26]	Maximal Strength and Isometric/Isokinetic Contractability “ “ “	↔	↔		EUM: 9 HC: 9
Nichols et al. [78]		↔	↔		EUM: 18 OC: 13
Elliott et al. [79]		↔	↔		EUM: 7 OC: 14
Ekenros et al. [76]		↔	↔		EUM: 9 OC: 8
Giacomoni et al. [77]	Muscular Power	↔	↔		EUM: 7 OC: 10
EUM Females across the Menstrual Cycle					
Source	Outcome	Follicular Phase	Late Follicular/Ovulatory Phase	Luteal Phase	n
Ekenros et al. [76]	Isometric/Isokinetic Contractability Muscular Strength	↔	↔	↑	9
Elliott et al. [79]		↔	-	↔	7
Romero-Moraleda et al. [15]		↔	↔	↔	13
Lebrun et al. [14]	Isometric/Isokinetic Contractability	↔	-	↔	16
Ekenros et al. [76]	“ “ “ “	↔	↔	↔	9
Sarwar et al. [16]		↔	↑	↔	10
Phillips et al. [81]		↑	↓	↑ *	22
Elliott et al. [79]		↔	-	↔	7
Montgomery et al. [82]		↔	-	↔	71
Romero-Moraleda et al. [15]	Muscular Power “	↔	↔	↔	13
Giacomoni et al. [77]		↔	-	↔	7
Ansdell et al. [71]	Neuromuscular Function	↓	↑	↑	15
Hormonal Contraceptive Users					
Source	Outcome	Pill Withdrawal Phase	Active Pill Phase		n
Ekenros et al. [76]	Maximal Strength “	↔	↔		8
Elliott et al. [79]		↔	↔		14
Ekenros et al. [76]	Isometric/Isokinetic Contractability “	↔	↔		8
Elliott et al. [79]		↔	↔		14
Sarwar et al. [16]		↔	↔		10
Rechichi et al. [80]	Muscular Power “	↓	↑		10
Giacomoni et al. [77]		↔	↔		10

A greater or increased measurement outcome is indicated by “↑”, while “↓” indicates a reduced or lower value, and “↔” indicates no significant difference or change. Dashes (-) indicate when an assessment was not performed during that cycle phase in that study. * In trained females only.

**Table 2:** Summary of inflammatory marker, blood lactate, and vascular responses to stimuli (exercise, initiation of contraception) across the menstrual cycle in eumenorrheic females as well as contraceptive users. All studies included recreationally active to active females.

Inflammatory Markers and BLA Across the Eumenorrheic Menstrual Cycle						
Source	Biomarker	Follicular Phase	Late Follicular Phase/Ovulation	Luteal Phase	Timepoint(s) of Significant Finding	n
Hackney et al. [97]	IL-6	↑	-	↓	0-hr POST, 24-hr POST, 72-hr POST	8
	CK	↑	-	↓	24-hr POST, 72-hr POST	
Romero-parra et al. [98]	IL-6	↓	↔	↑	2-hr POST > PRE, 24-hr POST, 48-hr POST	19
	CK	↔	↔	↔		
Markofski et al. [99]	CK	↓	-	↑	96-hr POST	16
Chaffin et al. [100]	IL-6	↔	-	↔		9
Tsampoukos et al. [101]	BLA	↔	↔	↔		8
Middleton et al. [102]	BLA	↔	-	↔		6
Lara et al. [103]	BLA	↔	↔	↔		13
Štefanovský et al. [104]	BLA	↔	-	↔		8
Cabre et al. [7]	BLA	↔	-	↔	No differences between pre-, half-way, post-, or 10-min post-BLA measures	19
Blood Lactate in Eumenorrheic Females Compared to Oral Contraceptive Users or Hormonal IUD Users						
Minahan et al. [105]	EUM	↔ Assessed on day 2–6 of cycle	-	-	No significant differences were observed between EUM and OC groups.	8
	OC Users	-	-	↔ Assessed on day 2–21 of active pill phase		8
Cabre et al. [7]	EUM	↔ Assessed on day 0–9 of cycle	-	↔ Assessed between 2 days after ovulation or 5 days before next predicted period	No significant differences were observed between EUM, OC, or Hormonal IUD groups or across phases.	19
	OC Users	- Assessed on days 0–7 of placebo pill	-	- Assessed on days 1–21 of active pill		21
	IUD Users	- Assessed on days 0–9 of cycle	-	- Assessed between 2 days after ovulation or 5 days before next predicted period		24
Vascular Outcomes across the Eumenorrheic Menstrual Cycle and Cycles with Contraceptive Use						
Source	Measurement Outcome	Follicular Phase/Inactive Pill Phase	Late Follicular Phase	Luteal Phase/Active Pill Phase	Timepoint(s) of Significant Finding	n
Williams et al. [106]	Endothelial function	↔	↑ *	↔	* Meta-analysis rated this finding with ‘very low’ certainty of evidence.	30 studies; n = 1363 EUM
	Smooth muscle function	↔	↔	↔		
Williams et al. [107]	Endothelial function Group:					
	Combined estrogen/progestin contraceptives	↑,↓,↔ *	-	↑,↓,↔ *	* Dependent on specific contraceptive	12 studies
	Progestin-only contraceptives	↔	-	↔		2 studies
	Smooth muscle function Group:					
	Combined estrogen/progestin contraceptives	↔	-	↔		10 studies
	Progestin-only contraceptives	↔	-	↔		2 studies
Blood flow in Eumenorrheic Females Compared to Oral Contraceptive Users or Hormonal IUD Users						
Minahan et al. [105]	EUM	↔ Assessed on day 2–6 of cycle	-	-	Skin blood flow plateaued in EUM from 7.5 min in the heated (35 °C condition), whereas in OC users, increased for 15 min	8
	OC Users	-	-	↑ Assessed on day 2–21 of active pill phase		8
Cabre et al. [7]	EUM	↔ Assessed on day 0–9 of cycle	-	↔ Assessed between 2 days after ovulation or 5 days before next predicted period	No significant differences were observed between EUM, OC, or Hormonal IUD groups or across phases.	19
	OC User	- Assessed on days 0–7 of placebo pill	-	- Assessed on day 1–21 of active pill		21
	IUD Users	- Assessed on days 0–9 of cycle	-	- Assessed between 2 days after ovulation or 5 days before next predicted period		24

A greater or increased measurement outcome is indicated by “↑”, while “↓” indicates a reduced or lower value, and “↔” indicates no significant difference or change. Dashes (-) indicate when an assessment was not performed during that cycle phase in that study.

## Data Availability

Data presented in this review were identified through PubMed and Google Scholar.
